# Hunting Activity Among Naturalistically Housed Chimpanzees *(Pan troglodytes)* at the Fundació Mona (Girona, Spain). Predation, Occasional Consumption and Strategies in Rehabilitated Animals

**DOI:** 10.3390/ani2030363

**Published:** 2012-08-14

**Authors:** Miquel Llorente, David Riba, Marina Mosquera, Mei Ventura, Olga Feliu

**Affiliations:** 1 Unitat de Recerca i Laboratori d’Etologia, Fundació Mona, Carretera de Cassà km4, Riudellots de la Selva, 17457 Girona, Spain; E-Mails: d.riba@fundacionmona.org (D.R.); recerca@fundacionmona.org (M.V.); o.feliu@fundacionmona.org (O.F.); 2 Institut Català de Paleoecologia Humana i Evolució Social, 43007 Tarragona, Spain; 3 Àrea de Prehistòria, Universitat Rovira i Virgili, 43002 Tarragona, Spain; E-Mail: marina.mosquera@urv.cat

**Keywords:** predatory behavior, hunting, cooperative behavior, captivity, welfare

## Abstract

**Simple Summary:**

Hunting is well documented in wild chimpanzees, but has rarely been documented in captive chimpanzees. At Fundació Mona Primate Rescue we have obtained evidence of five episodes of hunting in rehabilitated chimpanzees who had no previous experience of these types of behaviors. This demonstrated that they were able to perform this species-typical behavior in a naturalistic environment without learning it in the wild.

**Abstract:**

Predatory behavior in wild chimpanzees and other primates has been well documented over the last 30 years. However, as it is an opportunistic behavior, conditions which may promote such behavior are left up to chance. Until now, predatory behavior among captive chimpanzees has been poorly documented. In this paper, we present five instances providing evidence of predatory behavior: four performed by isolated individuals and one carried out in cooperation. The evidence of group predation involved the chimpanzees adopting different roles as pursuers and ambushers. Prey was partially eaten in some cases, but not in the social episode. This study confirms that naturalistic environments allow chimpanzees to enhance species-typical behavioral patterns.

## 1. Introduction

Hunting is relatively wide spread among many wild primates. Predation is mainly specific to arthropods and small vertebrates [1,2]. Predatory behavior in wild chimpanzees has been well documented over the last 30 years [3,4,5,6,7]. Studies have shown that it is a systematic behavior in different populations of chimpanzees [8], which may occur either individually or cooperatively. Cooperative hunting has social implications since it helps to maintain and reinforce social relationships among the individuals of a group. The most commonly hunted prey is red colobus (*Procolobus *sp.) at all long-term study sites: Gombe [9], Mahale [10], Ngogo [11], and Taï [12], among others. However, hunting episodes of small prey are also well documented [13]. Moreover, other primate species can be hunted by chimpanzees: *Galago senegalensis* [14], *Papio cynocephalus* [15], or *Erythrocebus patas* [16], among others. Furthermore, some authors have observed chimpanzees using tools in attempts to extract, capture and kill prey [17,18]. 

Predatory behavior in captive primates include reports of opportunistic individual predation of vertebrates by several species of the genus *Galago*, *Saguinus*, *Saimiri*, *Cebus*, *Lagothrix*, *Macaca*, *Mandrillus* and *Cercopithecus* [19]. This behavior has also been documented in other captive primates [20].

But surprisingly, evidence of predation is rarely documented in captive chimpanzees. The first evidence of this type of behavior came from recent reports [21]. These episodes were performed by isolated individuals, never those in groups and mainly focused on birds and small mammals. Recently, Ross and Lonsdorf [22] have evaluated the prevalence and nature of interactions between zoo-housed great apes and various North-American wildlife species. However, this study was carried out using a questionnaire of 14 items, but not fully describing the real behavioral observations of the great apes during the interactions toward those species. The lack of records of these kinds of episodes in captivity may be due to the fact that groups are rarely housed in large naturalistic enclosures, but instead they are located close to urban centers and the presence of native wildlife that may enter the enclosures is limited. 

We documented five episodes of well-recorded predatory behavior carried out by a group of chimpanzees originally coming from strongly humanized contexts and currently housed in a naturalistic environment. We consider that this report is important because the only other similar report described in the scientific literature was published by Videan and colleagues [21] at the Primate Foundation of Arizona, and it only referred to individual performances. At the Fundació Mona (FM), we recorded individual and social performances during hunting and in most cases they were associated with fast-moving animals. The question then remains whether non-experienced chimpanzees will exhibit predatory behavior similar to that of wild or other captive chimpanzees.

## 2. Material and Methods

### 2.1. Study Subjects and Facilities

Since 2000, the Fundació Mona (Girona, northeast Spain; 41°54'N, 2°49'E) has devoted itself to the rescue, recovery, rehabilitation, resocialisation and sheltering of primates that have been exploited or mistreated. The FM attempts to provide these primates with better living conditions through naturalistic environments and stable social groups in order to help the animals develop behavioral patterns typical of their species, promoting their well-being and welfare.

During the daytime the chimpanzees live in a naturalistic, main outdoor enclosure comprised of a total of 5,640 m^2^. The enclosure is divided into two areas: one is 2,420 m^2^ and the other is 3,220 m^2^, with a total division perimeter of 191 meters. The enclosure is surrounded by a steel fence and a 12 V electrified fence. Inside the enclosure are wooden platforms, towers and structures for climbing, resting and socializing. Two artificial water fountains provide drinking water *ad libitum*. The ground substrate is natural and characterized by a majority of Mediterranean and shoreline herbaceous vegetation. The vegetation is subject to seasonal changes and to the alteration of its physical environment by the chimpanzees. The following plant species are present: *Verbena officinalis*, *Conyza *sp., *Plantago lanceolada*, *Anthemis *sp., *Rumex obtusifolious*, *Portulaca oleracea*, *Verbascum blattaria, Taraxacum officinale* and *Polycarpon tetraphylum*, among others. The primates also have access to artificial termite mounds which are used for environmental enrichment. Occasionally, some native animals rest, feed or take up residence in or near the outdoor enclosure providing the chimpanzees an opportunity to hunt them. The most common species coming into contact with the naturalistic enclosure are: (1) Birds: House sparrow (*Passer domesticus*), Nightingale (*Luscinia megarhynchos*), Grey Heron (*Ardea cinerea*), European robin (*Erithacus rubecula*), Yellow-legged Gull (*Larus michahellis*), Black Kite (*Milvus migrans*), Hoopoe (*Upupa epops*), Common Blackbird (*Turdus merula*), European magpie (*Pica pica*), and Common Kestrel (*Falco tinnunculus*); (2) Mammals: European hedgehog (*Erinaceus europaeus*), European rabbit (*Oryctolagus cuniculus*), wood mouse (*Apodemus sylvaticus*), Brown rat (*Rattus norvegicus*), and European Mole (*Talpa europaea*); and (3) Amphibians and reptiles: grass snake (*Natrix natrix*), ladder snake (*Rhinechis scalaris*), Mediterranean tree frog (*Hyla meridionalis*), Perez’s frog (*Rana perezi*), Common toad (*Bufo bufo*), and the Iberian wall lizard (*Podarcis hispanica*); among others.

At the beginning of this study in January 2008, the sample group consisted of thirteen individuals, separated into two different groups: group A (Male group) and group B (Family group). The group of chimpanzees is formed of nine males and four females, but only 10 of them took part in the activities described here. They range from 4 to 51 years old. Between 2008 and 2011 some animals changed groups and in 2010 one of the subjects (Romie from the Family group) passed away due to natural causes. A new individual (Africa) was also introduced to group B in 2010. Table 1 shows the additional traits of these individuals: sex, age, place of birth and origin. The chimpanzees are fed juices, fresh fruit, fresh vegetables, boiled rice, nuts and other seeds four times per day and water is always available. During a routine day, animals forage and are fed food which is hidden or scattered throughout the naturalistic enclosure.

We conducted this research in accordance with all national and institutional guidelines for the care and management of primates established by FM.

**Table 1:** Additional traits of the Fundació Mona (FM) chimpanzees: sex, age, place of birth and origin.

Year arrived at Mona Foundation	Name	Sex	Birth date	Birth place	Background	Group	Participation in hunting
2009	África	Female	2000	Wild	Pet	B	YES
2002	Bongo	Male	2000	Captivity	Circus	B/A	YES
2001	Charly	Male	1989	Captivity	Circus, TV	A	YES
2003	Juanito	Male	2003	Captivity	Pet	B	YES
2001	Marco	Male	1984	Captivity	Circus, TV	A	YES
2004	Nico	Male	2001	Captivity	Pet	B	YES
2001	Romie	Female	1979	Wild	Breeding, Circus	B	YES
2004	Sara	Female	1998	Captivity	Pet, TV	B	YES
2005	Tico	Male	1985	Captivity	Pet, Zoo	B	NO
2001	Toni	Male	1983	Wild	TV, Zoo	A	NO
2003	Toto	Male	1956	Wild	Pet, Zoo	B	YES
2006	Víctor	Male	1982	Wild	Pet, Zoo	B	NO
2002	Waty	Female	1996	Captivity	Circus	B	YES

### 2.2. Behavioral Observations

Five cases of prey capture were observed between January 2007 and September 2011. The episodes of predation were observed during our ongoing Hand Laterality Research Project, which began in 2002 [23,24,25] and our on-going Rehabilitation and Resocialisation Project which began in 2006 [26,27]. All five predation episodes presented here were recorded while the researcher was present. This factor does not interfere with the results since all the chimpanzees are acclimated to the presence of the FM researchers. Three of the episodes were video and photographically recorded (episodes ID H1, H3 and H4). Two of the episodes were only photographically recorded (episodes ID H2 and H5). Data were recorded on an *ab libitum* basis during the predation events. Species of the animal prey, date, and time were recorded. Animals involved in the predation were also described. Regarding the H1 episode (social hunting), independent observers describe and interpret this observation the same way the original observer (MV) did. This event was videotaped, so it would be possible to test observer reliability by having three naïve observers (unaware of the original description and interpretation).

## 3. Results

Between 2007 and 2011, five occurrences of predatory behavior by the FM chimpanzees were directly recorded (Table 2). Each of the predatory events happened during the daily routine activities of the group and all of the cases below are described in chronological order. In the first episode, the entire group was involved in the predation (social hunting), while in the other episodes the predation incident was performed individually. Although some other predation incidents occurred during this five-year period, we only registered those in which one of the researchers was present from the beginning to the end of the episode (observed kills). During the study period we discovered some post-kill evidence (n = 10 species) but we discarded it because we did not have specific information about how it was produced. The post-kill evidence refers mainly to the following species: *Passer domesticus*, *Apodemus sylvaticus*, *Natrix natrix*, *Hyla meridionalis*, *Rana perezi*, and *Bufo bufo *(Table 3).

**Table 2:** Prey captured by FM chimpanzees between 2007 and 2011.

ID	Date	Species	Social/Individual	N° individuals involved	Predatory strategy	Prey consumption	System record	Chimpanzee group
H1	2007-01-19	*Oryctolagus cuniculus*	Social	4	Coordinated	Try	Video	B
H2	2008-05-24	*Turdus merula*	Individual	1	Individual	Yes	Photo	B
H3	2008-08-02	*Larus michaellis*	Social	3	Individual	Try	Video and photo	A
H4	2010-10-29	*Oryctolagus cuniculus*	Individual	2	Individual	Yes	Video and photo	A
H5	2011-09-17	*Oryctolagus cuniculus*	Individual	1	Individual	Try	Photo	B

**Table 3:** Native wildlife at FM with killing and post-killing evidence.

Species	Common name	Evidence of hunting	Chimpanzee group
*Passer domesticus*	House sparrow	Post-kill	A/B
*Luscinia megarhynchos*	Nightingale	No	
*Ardea cinerea*	Grey Heron	No	
*Erithacus rubecula*	European robin	No	
*Larus michahellis*	Yellow-legged Gull	Kill	A
*Milvus migrans*	Black kite	No	
*Upupa epops*	Hoopoe	No	
*Turdus merula*	Common Blackbird	Kill	B
*Pica pica*	European magpie	No	
*Falco tinnunculus*	Common Kestrel	No	
*Erinaceus europaeus*	European hedgehog	Post-kill	A/B
*Oryctolagus cuniculus*	European rabbit	Kill	A/B
*Apodemus sylvaticus*	Wood mouse	Post-kill	A/B
*Rattus norvegicus*	Brown rat	No	
*Talpa europaea*	European mole	Post-kill	A/B
*Natrix natrix*	Grass snake	Post-kill	A
*Rhinechis scalaris*	Ladder snake	Post-kill	A
*Hyla meridionalis*	Mediterranean tree frog	Post-kill	A/B
*Rana perezi*	Pere's frog	Post-kill	A/B
*Bufo bufo*	Common toad	Post-kill	A/B
*Podarcis hispanica*	Iberian wall lizard	Post-kill	A/B

### 3.1. Case 1 (H1)—19 January 2007. Social Predation of a Rabbit (Oryctolagus cuniculus)

**Figure 1:**
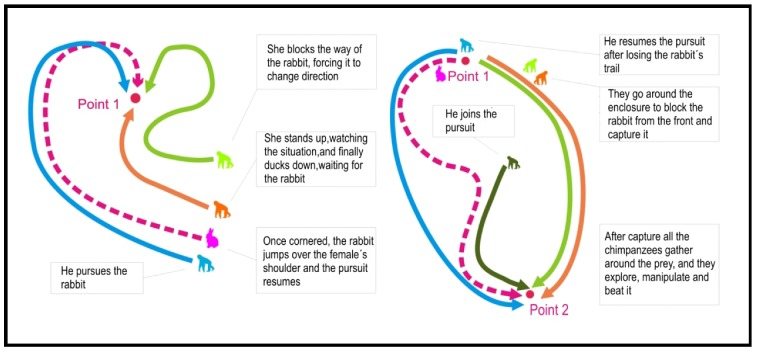
The hunting strategy employed by the FM chimpanzees during the H1 episode.

**Figure 2:**
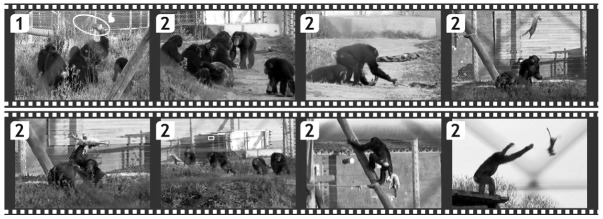
(**1**) *Slaughter*: Toto holds the rabbit by its hind legs and beats it to death against the ground. (**2**) *Exploration*: Toto discards the rabbit and juvenile and young chimpanzees start exploration and play behavior.

This episode was recorded on 19 January 2007. It started at 17:10. Four chimpanzees took an active part in the predation: two juvenile females, one young male and one juvenile male (Figure 1). A rabbit (*Oryctolagus cuniculus*) came into the enclosure by chance. It was finally captured by the four chimpanzees after two attempts in which the hunters took on different roles in the action. The first attempt was made by three chimpanzees (one juvenile male and two juvenile females). The male (Bongo) pursued the prey while the females (Sara and Waty) positioned themselves for an ambush to block the rabbit’s escape route and to capture the animal (Figure 1). The second attempt was performed by all four chimpanzees (one young male, one juvenile male and two juvenile females). One of them (Juanito) performed the role of pursuer while the others (Waty, Sara and Bongo) set up an ambush in a semicircular position, blocking and capturing the prey. Once the hunting episode was finished, the other three chimpanzees joined the group: one adult female (Romie), one adult male (Toto) and one young male (Nico). All the individuals gathered around the rabbit, which was taken by its hind legs by the oldest chimpanzee (Toto) and beaten once against the ground with one fatal blow. Immediately, a sequence of exploration and brutal play behaviors started among the five juvenile and young individuals (Figure 2). The prey was not consumed, although some individuals tried to bite it.

### 3.2. Case 2 (H2)—24 May 2008. Individual Predation of a Common Blackbird (Turdus merula)

**Figure 3:**
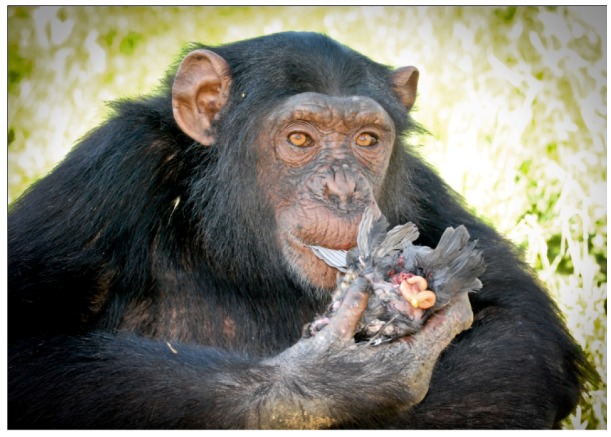
Predation of a *Turdus merula* (episode H2) by Nico.

This episode was recorded on 24 May 2008 between 15:46 and 15:59. Only one chimpanzee (Nico, male adolescent) took part in the episode. No other group individual was present during the predation episode and the prey manipulation. The capture of the prey was done opportunistically while the prey was on the ground. The prey was a young individual from the *Turdus merula* species. Nico hunted the prey with his hands and killed it with a blow. For five minutes Nico manipulated the prey in a playful manner. Later, using his mouth, he tore open the prey’s abdomen and ingested part of the guts, ignoring the wings, head and lower extremities (Figure 3). After the episode the prey was left in the same place.

### 3.3. Case 3 (H3)—2 August 2008. Individual Predation of a Yellow-Legged Gull (Larus michahellis)

**Figure 4:**
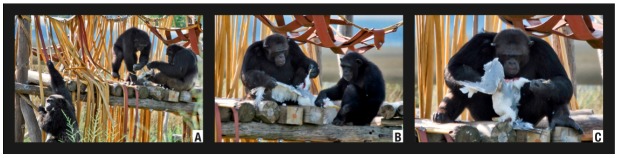
Predation of a *Larus michahellis* (Episode H3) by Male Group.

The episode was recorded on 2 August 2008 between 17:22 and 17:38. Three individuals were involved in the hunting episode. An adult *Larus michahellis* specimen remained in the artificial pool of the enclosure. Charly (Group A, adult male) captured the prey with his hands. We neither recorded the killing strategy nor who was the actual killer. Immediately afterwards Marco (*Alpha *male) took hold of the already dead prey and carried it to one of the enclosure towers accompanied by Bongo (adolescent male) and by Charly (Figure 4(a)). Charly left the tower after one minute. Bongo and Marco remained together until the end of the episode. From 17:25 to 17:27, Marco examined the prey. During this time, Bongo simply observed without taking part. At 17:28, Bongo struck the head of the prey with his hands. At 17:29 Marco began ripping off one of the lower limbs of the prey (Figure 4(b)). At 17:32 he bit the limb of the prey to pull it apart (Figure 4(c)). Marco ended up abandoning the prey in the tower structure.

### 3.4. Case 4 (H4)—29 October 2010. Individual Predation of a Rabbit (Oryctolagus cuniculus)

**Figure 5:**
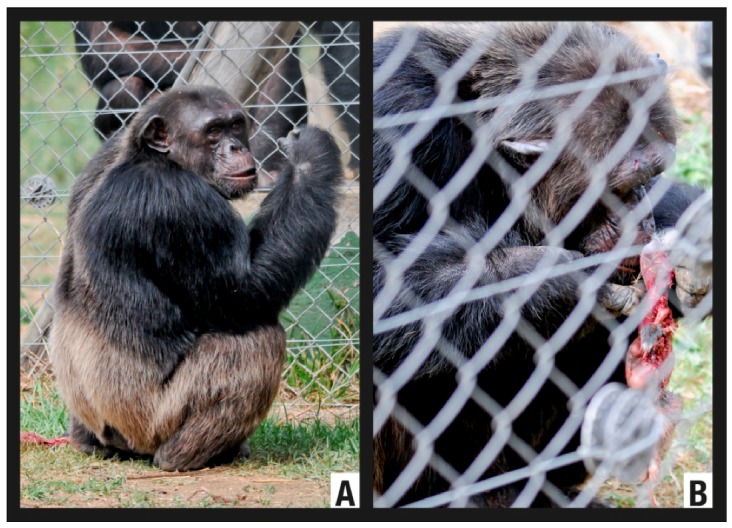
Predation of an *Oryctolagus cuniculus* (Episode H4) by Marco.

The episode was recorded on 29 October 2010 between 14:45 and 14:55. Marco (Group A, adult male) was involved in the episode but we neither detected the predation strategy, the exact moment of predation, nor exactly how he hunted. We photographically recorded the episode from 14:49 (Figure 5(a)). At that moment, the prey already appeared ripped apart with parts of its body missing, presumably ingested (Figure 5(b)). At 14:55, the prey was abandoned on the enclosure substrate. 

### 3.5. Case 5 (H5)—17 September 2011. Individual Predation of a Rabbit (Oryctolagus cuniculus)

**Figure 6:**
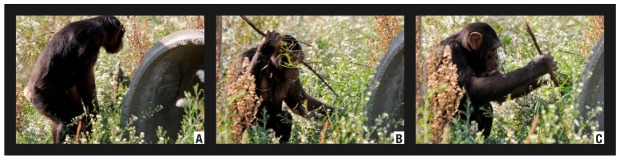
Predation of an *Oryctolagus cuniculus* (Episode H5) by Family Group. Tool use manipulation of the prey.

**Figure 7:**
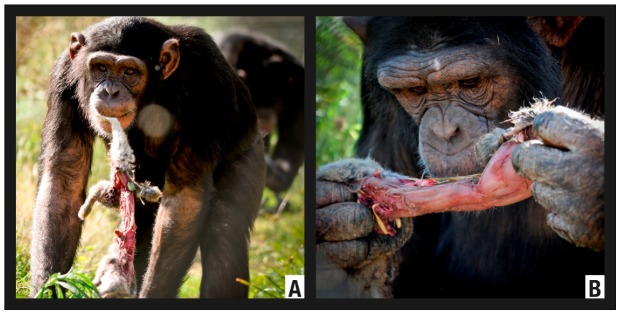
Predation of an *Oryctolagus cuniculus* (Episode H5) by Family Group. Transport and consumption of the prey.

An episode was registered on 17 September 2011 between 12:20 and 12:30. Juanito (Group B, male adolescent) was the subject most implicated in the episode but we neither detected his predation strategy, the exact moment of predation, nor exactly how he hunted. Two other individuals (Nico, male adolescent) and (Africa, female adolescent) played secondary roles in the episode. At 12:22, we started to photographically record the hunting episode. At 12:23, the prey was placed on the ground and Juanito took hold of a wooden stick to use it as a spear and began to repeatedly stab the rabbit with it (Figure 6). At 12:24 Nico rejoined Juanito. At that moment, Juanito approached the perimeter of the enclosure and threw the rabbit against the electrified fence. At 12:26 he returned transporting the prey in his mouth (Figure 7(a)). The abdomen of the rabbit was totally open with the intestines missing. One of the lower limbs was missing its muscular mass (possibly due to ingestion) and it was possible to see exposed bone. At 12:30 Juanito returned holding the prey and brought it up to his mouth, tasting it but without totally ingesting it (Figure 7(b)). Finally, the prey was discarded on the ground. 

## 4. Discussion

Predation on fast-moving animals (*i.e.*, rabbits) or large birds (*i.e.*, seagulls) has not been documented in captive chimpanzees despite several decades of observation of these animals in captive and naturalistic settings. The only report about hunting behavior in captive chimpanzees was published by Videan and colleagues several years ago [21]. Even rehabilitated animals and non-experienced subjects, when given the opportunity will hunt and occasionally partially consume prey items. Although wild chimpanzees rarely capture an animal without eating it, in both the wild and in captivity, some individuals (such as at FM) hunt but do not eat prey [28]. Once they have killed, some wild chimpanzees prefer to play with rather than consume their prey [29] as we reported in all five episodes at Mona. Wild bonobos (*Pan paniscus*) also showed similar exploration and grooming following their capture of infant monkeys which were handled and manipulated like dolls [30]. At FM the prey was not eaten entirely but partially consumed [14]. According to the roles, juveniles and adolescent individuals showed more interest in the prey, although some adult subjects participated in the hunting events as well. In wild settings the active role of males during the “hunting but not eating” episodes was not found [28]. In the case of bird predation we found some similarities between FM and wild chimpanzees. In Bossou [28] and in Mahale [31] the catching of birds is mainly solitary and opportunistic, not requiring social hunting, and the performance and strategy tend to be repeated in subsequent occasions. From our point of view, the present findings shed light on the behavioral recovery and diversity of rehabilitated chimpanzees and on the ability of these animals to develop species-typical behaviors in these types of naturalistic enclosures, although unusual in that they did not eat much of the meat. Furthermore, we hypothesize that predation could be a form of boredom alleviation despite the extensive methods of environmental enrichment engaged by Fundació Mona [27,32,33,34,35]. In fact, although with a decreasing trend, inactivity is the most frequent behavior in the study sample [36]. 

It is worth emphasizing that the H1 episode is the only social (likely cooperative) hunting event documented in a naturalistic environment to date. The FM chimpanzees mainly come from isolated environments, born in captivity or captive since youth. Therefore, either by mutual learning during their time spent at the FM or by innate nature, hunting seems to be both common and frequent in naturalised environments. Another plausible interpretation can be found for the social hunting episode. Captive chimpanzees can intercept moving targets (*i.e.*, catch them by moving to a spot where their target will predictably move). So, the behavior described here as cooperative “ambushing” might instead be individual efforts to catch the rabbit independently by interception. 

Furthermore, two other questions emerge from the evidence described here. Firstly, concerning the roles established by the chimpanzees in social hunting episode (H1), some individuals pursued the prey while others set up ambushes to block and capture it. These roles directly indicate spontaneous social organization for some tasks. However, hunting in FM chimpanzees appears mostly solitary and associated with males and adolescent subjects. Secondly, in the wild predatory behavior usually consists of both hunting and consuming. However, the animals involved in episodes H2, H3, H4 and H5 were partially eaten, but the rabbit from the social episode (H1) was not eaten at all. Therefore, it seems that hunting and consuming may occur and function separately. 

Also, it is worth noting that in H1, the chimpanzee that took charge and beat the prey before any of the other individuals was the oldest individual (Toto) who did not take part in the hunting episode. Further research will study whether the other chimpanzees implicitly accepted his higher authority in terms of his age. If so, and similar to the wild, predation in captive chimpanzees may be related to the maintenance and demonstration of social relations.

This study also has some important implications for the surveillance of outdoor enclosures (and other types of facilities) and the potential for disease or introduction of parasitic infection by captured prey such as: *Cheyletiellosis dermatitis *[37], *Fancisella tularensis *[38,39], *Salmonella *sp. [40,41] or *Entamoeba histolytica* [42], among others.

## 5. Conclusions

We emphasize the following conclusions: (1) predatory events are an important element of chimpanzee’s natural behavioral repertoire and should be considered as part of the design of enriched environments; (2) co-operative hunting roles for large prey are an emergent feature of chimpanzee social structures even in small groups; (3) predation may serve important social functions or alleviate boredom; and (4) females are active participants in hunting episodes, challenging some of the hunter/gatherer hypotheses regarding primate and hominin evolution.

Finally, we want to underscore the importance of naturalistic outdoor enclosures to enhance species-typical behavioral patterns and welfare [43,44]. This evidence highlights the significance of comparing the behavior of wild and semi-captive chimpanzees, particularly where hunting is concerned. We consider that further research and monitoring is needed to systematically assess the strategy, consumption and performance of predatory behavior in captive rehabilitated chimpanzees.
